# High-Frequency Stimulation of Ventral CA1 Neurons Reduces Amygdala Activity and Inhibits Fear

**DOI:** 10.3389/fnbeh.2021.595049

**Published:** 2021-03-09

**Authors:** Jalina Graham, Alexa F. D’Ambra, Se Jung Jung, Yusuke Teratani-Ota, Nina Vishwakarma, Rasika Venkatesh, Abhijna Parigi, Evan G. Antzoulatos, Diasynou Fioravante, Brian J. Wiltgen

**Affiliations:** ^1^Center for Neuroscience, University of California, Davis, Davis, CA, United States; ^2^Department of Psychology, University of California, Davis, Davis, CA, United States; ^3^Department of Neurobiology, Physiology and Behavior, University of California, Davis, Davis, CA, United States; ^4^Department of Plant Sciences, University of California, Davis, Davis, CA, United States

**Keywords:** learning, memory, optogenetics, context fear, mice, hippocampus

## Abstract

The hippocampus can be divided into distinct segments that make unique contributions to learning and memory. The dorsal segment supports cognitive processes like spatial learning and navigation while the ventral hippocampus regulates emotional behaviors related to fear, anxiety and reward. In the current study, we determined how pyramidal cells in ventral CA1 respond to spatial cues and aversive stimulation during a context fear conditioning task. We also examined the effects of high and low frequency stimulation of these neurons on defensive behavior. Similar to previous work in the dorsal hippocampus, we found that cells in ventral CA1 expressed high-levels of c-Fos in response to a novel spatial environment. Surprisingly, however, the number of activated neurons did not increase when the environment was paired with footshock. This was true even in the subpopulation of ventral CA1 pyramidal cells that send direct projections to the amygdala. When these cells were stimulated at high-frequencies (20 Hz) we observed feedforward inhibition of basal amygdala neurons and impaired expression of context fear. In contrast, low-frequency stimulation (4 Hz) did not inhibit principal cells in the basal amygdala and produced an increase in fear generalization. Similar results have been reported in dorsal CA1. Therefore, despite clear differences between the dorsal and ventral hippocampus, CA1 neurons in each segment appear to make similar contributions to context fear conditioning.

## Introduction

The hippocampus can be divided into distinct segments that make unique contributions to learning and memory (Fanselow and Dong, 2010). The dorsal segment supports cognitive processes like spatial learning and navigation via interactions with the entorhinal, parahippocampal, and retrosplenial cortices (Moser and Moser, 1998; Cenquizca and Swanson, 2006, 2007; Strange et al., 2014; Moser et al., 2017). The ventral hippocampus (VH), in contrast, regulates emotional behavior through its connections with the amygdala, nucleus accumbens, lateral hypothalamus, BNST and medial prefrontal cortex (Cenquizca and Swanson, 2006, 2007; Hoover and Vertes, 2007; Jimenez et al., 2018). Despite these differences, the dorsal and ventral hippocampus share some important properties. They have the same basic architecture and intrinsic organization (tri-synaptic loop) and neurons in both regions respond to spatial cues (e.g., place cells) (Ishizuka et al., 1990; Kjelstrup et al., 2008; Strange et al., 2014). These parallels suggest that similar computations may be carried out in the DH and VH during cognitive and emotional learning.

The integration of spatial and emotional information depends on interactions between the DH and VH. For example, during context fear conditioning, animals learn to associate a novel environment with aversive footshock. Encoding this relationship requires spatial information from the DH to be transmitted to the basal nucleus of the amygdala (BA) via the VH (Fanselow and Dong, 2010; Xu et al., 2016). However, neurons in the VH do not act as passive relays; their activity is strongly modulated by emotional states like fear and anxiety, which is not typically the case in the DH (Ciocchi et al., 2015; Jimenez et al., 2018). Consistent with this fact, lesions of the VH reduce stress hormone release and anxiety-related behaviors while damage to the DH does not (Kjelstrup et al., 2002). Place cells in the VH are also distinct; they have large, overlapping place fields that encode behaviorally-relevant contexts as opposed to precise spatial locations (Komorowski et al., 2013). Based on these findings, we hypothesized that dorsal (dCA1) and ventral (vCA1) CA1 neurons will respond to different stimuli during context fear conditioning. Specifically, we predicted that neurons in dCA1 would primarily respond to the spatial context while cells in vCA1 would be more responsive to footshock.

To examine our hypothesis, we quantified immediate-early gene expression (IEG) in vCA1 neurons after spatial exploration or emotional learning. For the former, mice were exposed to a novel environment and for the latter, mice underwent context fear conditioning. We found that vCA1 neurons were strongly activated by the novel environment but, surprisingly, c-Fos expression did not increase further when the context was paired with shock. Neurons in dCA1 have been shown to respond in the same way under similar conditions (Radulovic et al., 1998; Lovett-Barron et al., 2014). Next, we stimulated vCA1 neurons that project to the BA to determine if defensive behaviors could be induced after context fear conditioning. We found that high-frequency stimulation (20 Hz) disrupted freezing and led to feed-forward inhibition of principal cells in the BA. In contrast, low frequency stimulation (4 Hz) increased fear generalization and did not inhibit the BA. Similar results have been reported when dCA1 neurons are stimulated at low frequencies (Ryan et al., 2015). These data suggest that dorsal and ventral CA1 make similar contributions to context fear conditioning despite the functional differences between these regions.

## Results

### Context Learning Activates vCA1 Neurons That Project to the Basal Amygdala

**FIGURE 1:**
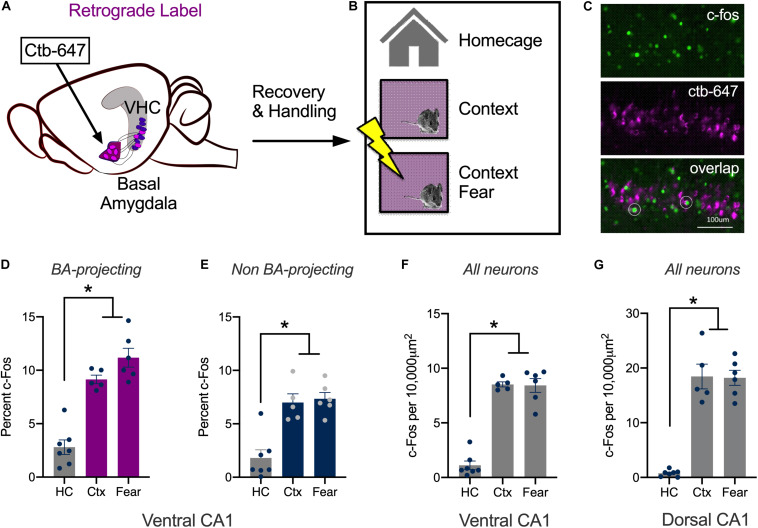
Context learning activates vCA1 neurons that project to the basal amygdala. **(A–C)** Experimental design. **(A)** Ctb (magenta) was injected into the BA. **(B)** Mice were sacrificed 90 min after exploring a novel context (Ctx) or undergoing context fear conditioning (Fear). Control animals remained in their homecages (HC). **(C)** Example histology showing c-Fos staining in green, Ctb labeling in magenta and overlap in BA-projecting neurons (right). **(D,E)** The percentage of BA-projecting (Ctb+) and non-projecting (Ctb−) vCA1 neurons that expressed c-Fos in HC, Ctx and Fear groups. **(F,G)** The number of neurons in ventral and dorsal CA1 that expressed c-Fos in HC, Ctx and Fear groups. Data are presented as mean ± SEM. *p < 0.05.

The DH responds to spatial and contextual cues while amygdala neurons respond strongly to emotional stimuli like footshock (O’Keefe and Dostrovsky, 1971; O’Keefe and Speakman, 1987; Radulovic et al., 1998; Pelletier et al., 2005; Barot et al., 2009; Wolff et al., 2014; Beyeler et al., 2018; Tanaka et al., 2018). The current experiment determined how vCA1 neurons respond to these stimuli using c-Fos as a proxy for neural activity and plasticity. To do this, we compared changes in c-Fos expression after mice were exposed to a novel environment or underwent context fear conditioning. Expression was quantified in neurons that send direct projections to the BA and those that do not. To identify the former, the retrograde tracer ctb-647 was infused into the BA prior to conditioning (Figure 1A). On the training day, control mice were left in their home cages (HC, *n* = 7). The context group (Ctx, *n* = 5) explored a novel environment for 5 min and the context + shock group underwent contextual fear conditioning (Fear, *n* = 6) (Figure 1B). Fear conditioning consisted of two footshocks (2 s, 0.3 mA, separated by 1 min) that were delivered after a 3 min exploration period. Ninety-minutes after training, the animals were sacrificed, and their brains fixed for c-Fos immunohistochemistry (Figure 1C). Compared to the control group, there was an increase in the number of c-Fos positive vCA1 neurons in mice that explored the novel environment or underwent context fear conditioning. The size of this increase was similar for the experimental groups and observed both in vCA1 neurons that project to the BA (Ctb + neurons) as well as those that do not (Ctb- neurons). Interestingly, a higher percentage of Ctb + cells expressed c-Fos than Ctb- cells in all groups (Figures 1D,E) [Repeated Measures ANOVA, Main effect of Group, *F*(2, 15) = 42.99, *p* < 0.0001; Main effect of Cell type, *F*(1, 15) = 22.93, *p* = 0.0002; No Group × Cell type interaction *F*(2, 15) = 3.115, *p* = 0.0739; Bonferroni *post hoc* tests, Control vs. Context (*p* < 0.0001), Control vs. Context + Shock (*p* < 0.0001), Context vs. Context + shock (*p* = 0.5778)].

While the expression of c-Fos did not increase significantly in the fear group compared to the context group, there was a trend in this direction. The lack of a difference could be due to the fact that we used F1 hybrids (C57BL/6 × 129S6) rather than the more commonly used C57BL/6 strain. Hybrids acquire more fear than C57s and can be trained with lower shock intensities and fewer trials (Owen et al., 1997; Balogh and Wehner, 2003). To determine if this difference affected our results, we fear conditioned a group of C57BL/6 mice with three, 0.75 mA shocks (Fear, *n* = 7) and compared them to animals that only explored the context (Ctx, *n* = 9) Ctb was once again infused into the BA to label vCA1 neurons that project to this region. Similar to the data collected in hybrid mice, c-Fos expression was similar in fear conditioned animals and those that explored the context. This was true both in Ctb + neurons (Ctx mean = 11.17%, SEM = 0.7387; Fear mean = 11.57%, SEM = 0.9617) and in Ctb- cells (Ctx mean = 7.932%, SEM = 0.9590; Fear mean = 6.113%, SEM = 0.5846). In addition, the overall amount of c-Fos expression was once again higher in vCA1 neurons that project to the BA (Ctb + mean = 11.37%, SEM = 0.850) compared to those that do not (Ctb- mean = 7.02%, SEM = 0.771) [Repeated Measures ANOVA, Main effect of cell type (Ctb + /Ctb−), *F*(1, 14) = 16.55, *p* < 0.0012; No effect of group, *F*(1, 14) = 1.698, p = 0.2135; No group × cell type interaction *F*(1, 14) = 1.077, *p* = 0.3170] (Data not shown).

Together with previous work, these results demonstrate that novel environments strongly activate pyramidal neurons in dorsal and ventral CA1. Pairing the environment with shock does not further increase activity in either of these subregions (measured via c-Fos), as it does in subcortical areas like the amygdala (Milanovic et al., 1998; Radulovic et al., 1998; Barot et al., 2009). To ensure we could replicate the results of prior studies done in dCA1, we quantified c-Fos expression in this region and compared it to vCA1 in the same animals. For these analyses, single scan planes were taken from each area and the number of c-Fos + neurons were counted per 10,000 um^2^. The results obtained with this methodology were similar to the vCA1 data described above; c-Fos expression increased in the context and fear conditioning groups compared to homecage controls and these conditions did not differ from one another. The same pattern was found in dCA1, although in this region, the total number of c-Fos + cells was higher than we observed in vCA1 (Figures 1F,G) [Repeated Measures ANOVA, Main effect of Group *F*(2, 15) = 120.3, *p* < 0.0001; Main effect of Region *F*(1, 15) = 55.64, *p* < 0.0001; Group × Region interaction *F*(2, 15) = 16.88, *p* = 0.0001; Bonferroni *post hoc* tests, in both dCA1 and vCA1, HC vs. Context (*p* < 0.0001), HC vs. Fear (*p* < 0.0001) Context vs. Fear (*p* > 0.9999); dCA1 vs. vCA1, HC vs. HC (*p* > 0.9999), Fear vs. Fear (*p* < 0.0001) Context vs. Context (*p* > 0.9999)].

We should note that vCA1 neurons activated during context exploration may also respond to footshock. If that were the case, it could be difficult to find differences in c-Fos expression between fear conditioned animals and mice that were exposed to the context. This issue could be addressed in future studies by labeling context responsive cells and footshock activated neurons with different IEGs (Barot et al., 2009). Single-unit recordings and Ca^2+^ imaging could also be used to examine the activity of individual vCA1 neurons during exploration and fear conditioning. An advantage of these tools is that precise firing patterns can be obtained and compared across different experimental conditions.

### *In vitro* Stimulation of vCA1 Neurons With ChETA

**FIGURE 2:**
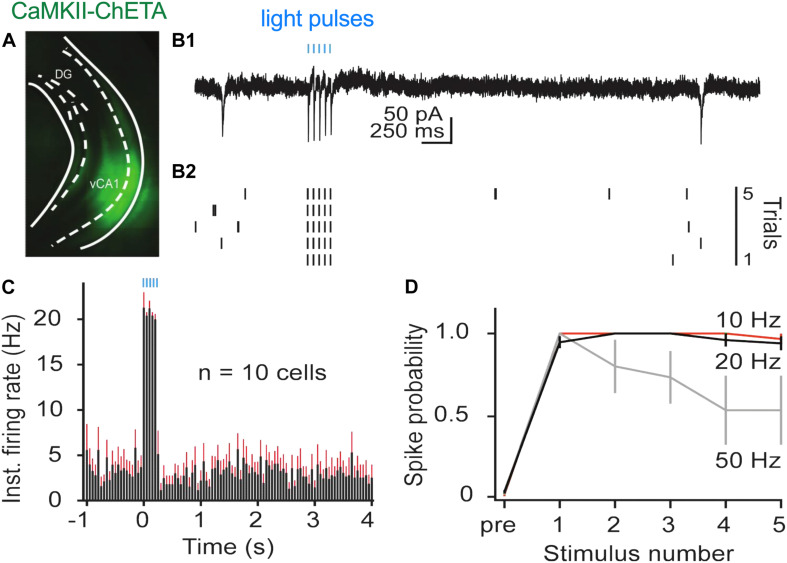
*In vitro* stimulation of vCA1 neurons with ChETA**. (A)** Expression of ChETA-EYFP in ventral/intermediate CA1. **(B)** *Ex vivo* cell-attached recording from a representative ChETA-expressing vCA1 pyramidal neuron in response to five 10 ms light pulses at 20 Hz (blue lines). **(B1)** Single example trial. **(B2)** Raster plot of 5 trials. **(C)** Average instantaneous firing rate (in spikes/s) for vCA1 pyramidal neurons (*N* = 10; bins: 50 ms, black: average firing rate, red: SEM). **(D)** Average spike probability for 3 photostimulation frequencies (10, 20, and 50 Hz). Data are presented as mean ± SEM.

High-frequency stimulation (20 Hz) in dCA1 does not induce freezing after fear conditioning like it does in the dentate gyrus (DG) and CA3 (Ramirez et al., 2013; Ryan et al., 2015; Oishi et al., 2019). This may be the case because dCA1 does not send a direct projection to the ventral segment of the hippocampus like DG and CA3 (Fricke and Cowan, 1978; Swanson et al., 1978; Ishizuka et al., 1990). To examine this idea, we used ChETA to stimulate pyramidal neurons in vCA1 after context fear conditioning. We first identified optimal stimulation parameters in hippocampal slices by infusing AAV5-ChETA-EYFP into the VH and recording from vCA1 neurons 2–3 weeks later. Recordings were performed using a cell-attached patch configuration (see section “Materials and Methods” for details) while stimulating with 488 nm light at 10, 20, or 50 Hz (Figure 2). As observed in dCA1, pyramidal cells in vCA1 could easily follow 10 and 20 Hz optogenetic stimulation across multiple trials (Figure 2D). At these frequencies, the spike probability for light pulses 2–5 was close to 1 and not significantly different from the spike probability for the first pulse (permutation test for pulses 2–5 compared to pulse 1: 10 Hz: *p* = 0.82, *p* = 0.82, *p* = 0.82, *p* = 0.20; 20 Hz: *p* = 0.89, *p* = 0.88, *p* = 0.62, *p* = 0.43). In contrast, when the same neurons were stimulated at 50 Hz, they responded reliably only to the first light pulse (average spike prob. ± SEM: 1.0 ± 0.00). The firing probability to subsequent stimuli progressively decreased and was significantly reduced by pulse 4 (Figure 2D) (permutation test for pulses 2–5 compared to pulse 1: 50 Hz: *p* = 0.17, *p* = 0.09, *p* = 0.01, *p* = 0.001).

### *In vivo* Stimulation of vCA1 Neurons With ChETA

**FIGURE 3:**
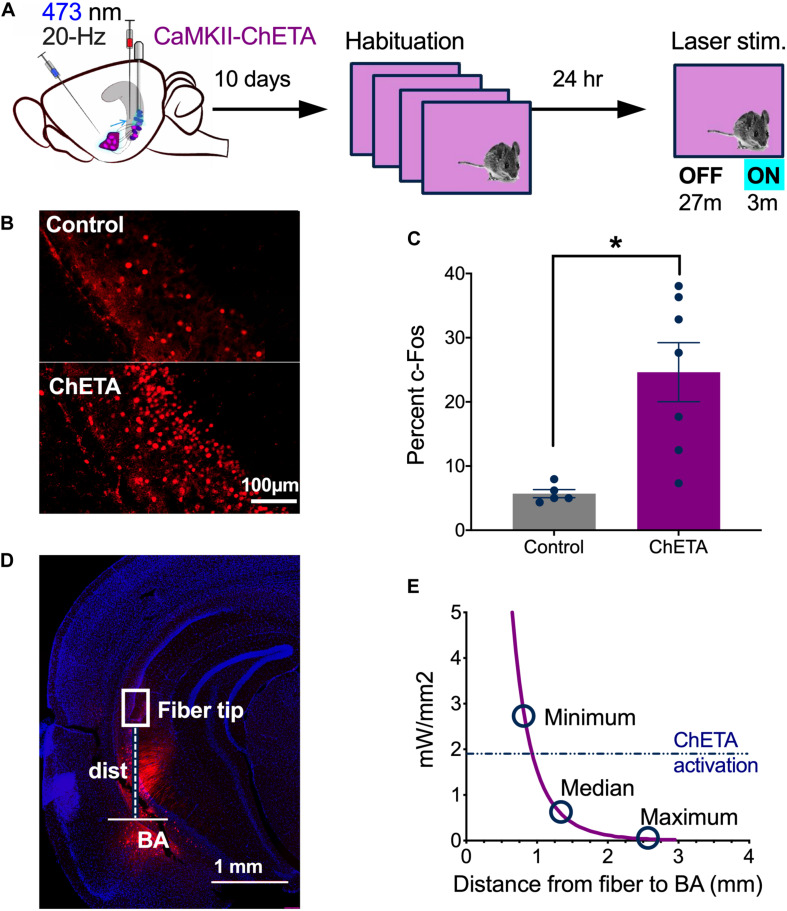
*In vivo* stimulation of vCA1 neurons with ChETA. **(A)** Experimental design. **(B)** Examples of c-fos expression in a control animal (top) and a mouse that had vCA1 pyramidal neurons stimulated with ChETA (bottom). **(C)** The percentage of c-Fos positive vCA1 neurons in Control and ChETA groups. **(D)** Image showing an example of AAVrg-ChETA-mCherry expression in vCA1 and BA and how distance was measured between the optic fiber tip and the BA. **(E)** Predicted irradiance values were plotted against distance from the optic fiber tip. The minimum, median and maximum fiber tip to BA distance values are marked on the curve. The dashed line indicates the threshold at which ChETA responds to light stimulation with less than 10% fidelity (Berndt et al, 2011). Data are presented as mean ± SEM. *p < 0.05.

Next, we confirmed that 20 Hz laser stimulation activated vCA1 pyramidal neurons *in vivo* (Figure 3). Mice received infusions of CaMKII-ChETA-EYFP (*n* = 7) or a control virus (CaMKII-EYFP, *n* = 5) and 10 days later were habituated to a novel environment for 4 days (30 min each day) to reduce c-Fos expression (Hess et al., 1995). On Day 5, they were returned to the same context and received 3 min of laser stimulation after a 27 min baseline period (Figure 3A). Ninety-minutes after this session, the animals were sacrificed, and their brains fixed for c-Fos immunohistochemistry. We found a significant increase in the number of c-Fos + neurons in the ChETA group compared to control animals, indicating that 20 Hz stimulation strongly activated vCA1 neurons (Figures 3B,C) (Two-tailed unpaired *t*-test, *p* = 0.0066, *t* = 3 df = 10). While the majority of ChETA + neurons were found in vCA1, we also observed some expression in the BA. To determine if light delivered to vCA1 could activate these cells directly, we measured the distance between each of our fiber tips and the BA (Figure 3D). These data (minimum, median, and maximum distances) were then plotted against the predicted decay in laser power observed when light passes through tissue (Figure 3E) (Stanford predicted irradiance tool), (Yizhar et al., 2011). The dashed line on this figure (0.90–0.95 mm) indicates the distance at which blue light stimulation (10 mW, 20 Hz) fails to produce an action potential >90% of the time in ChETA + neurons (Berndt et al, 2011). We found that 94% of our fibers fell beneath this line, making it unlikely that light stimulation in vCA1 would activate BA neurons directly.

### High-Frequency Stimulation of vCA1 Neurons Impairs the Expression of Context Fear

**FIGURE 4:**
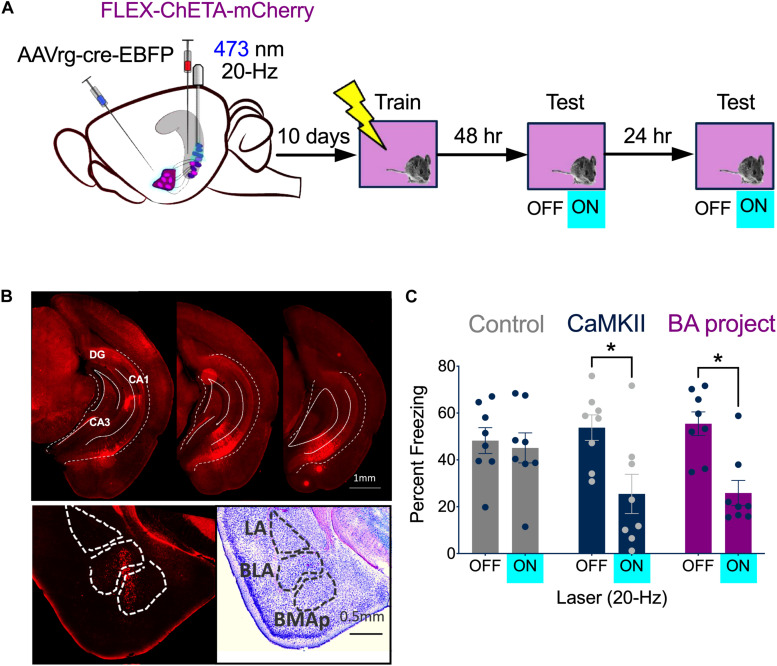
High-frequency stimulation of vCA1 neurons impairs the expression of context fear. **(A)** Experimental design. Stimulation groups received infusions of CaMKII-ChETA-EYFP or FLEX-ChETA-EYFP into the VH. Control groups received infusions of CaMKII-EYFP or FLEX-EYFP into the VH. To target amygdala projecting vCA1 neurons, mice that received FLEX viruses also had AAVrg-Cre-EBFP infused into the BA. **(B)** Expression of FLEX-ChETA-mCherry in vCA1 (top); Immunostaining for Cre in the BA (bottom left) and an adjacent Nissl section (bottom right). **(C)** Average freezing during baseline (OFF) and 20 Hz stimulation epochs (ON) during the memory retrieval tests. Data are presented as mean ± SEM. *p < 0.05.

Based on our recording and c-Fos results, we decided to stimulate vCA1 pyramidal neurons at 20 Hz after context fear conditioning. Our initial plan was to activate neurons that expressed c-Fos during training (i.e., engram/memory cells) using TetTag mice from Jackson labs (stock no. 008344). However, we observed a significant amount of non-specific labeling in these mice compared to our original fos-tTA line (Tayler et al., 2013; Tanaka et al., 2014; Nakazawa et al., 2016; Wilmot et al., 2018; Crestani et al., 2019). Therefore, instead of targeting c-Fos + cells, we stimulated vCA1 neurons that project to the BA. To do this, AAVrg-EBFP-Cre was injected into the BA and FLEX-ChETA-mCherry virus was infused into the VH (BA project, *n* = 8) (Figure 4A, left). Bilateral optic fibers were implanted directly over vCA1. Histological analyses confirmed that ChETA-mCherry was expressed in vCA1 neurons (Figure 4B, top) and Cre expression was restricted to cells in the BA (Figure 4B, bottom). A second group received infusions of CaMKII-ChETA-EYFP into vCA1 (All vCA1 *n* = 8) to examine the effects of non-selective stimulation on freezing. Control groups received infusions of AAV-CaMKII-EYFP into vCA1 (*n* = 4) or combined injections of AAVrg-EBFP-Cre into BA and FLEX-tdTomato in vCA1 (*n* = 4).

Following recovery from surgery, animals were handled and habituated to the optic fiber cable for 5 days and then were trained on context fear conditioning. Training consisted of a 3 min baseline period followed by 2 shocks (0.3 mA, 2 s duration) delivered 1 min apart. Two days later, the mice were placed back in the training environment to assess context fear memory. The test began with a 3 min baseline period that was followed by 3 min of stimulation with blue light (473 nm, 10 mW, 20 Hz). Mice received an identical test 24 h later (Figure 4A, right). During the baseline period, freezing was similar for all groups (Figure 4C). When 20 Hz laser stimulation was delivered to vCA1, freezing decreased significantly in the ChETA groups, but not in control animals [Repeated measures 2-way ANOVA, Stimulation × Group interaction *F*(2, 21) = 5.88, *p* = 0.0093; Bonferroni *post hoc* tests, Control On vs. Off (*p* > 0.9999), All vCA1 On vs. Off (*p* = 0.0005) BA- projecting vCA1 On vs. Off (*p* = 0.0003)]. The size of this decrease was similar whether all vCA1 pyramidal neurons were stimulated or just those that project to the BA [No group x laser interaction, *F*(1,14) = 0.01590, *p* = 0.9014]. These results demonstrate that context fear is not enhanced by high-frequency stimulation of vCA1 neurons. Therefore, the inability of 20 Hz stimulation to induce freezing in dCA1 is not due to the fact that this region lacks direct projections to the VH or the amygdala (Ramirez et al., 2013; Wilmot et al., 2019; Krueger et al., 2020). To explain our results, we next examined the effects of vCA1 stimulation on the activity of principal cells in the BA.

### High Frequency Stimulation of vCA1 Terminals Inhibits Principal Cells in the BA

**FIGURE 5:**
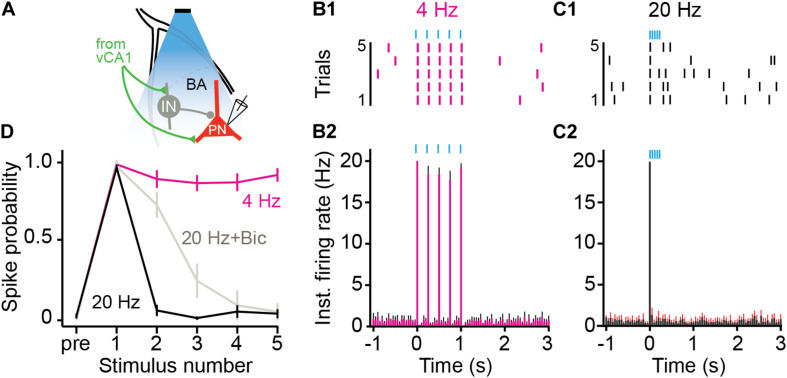
High frequency stimulation of vCA1 terminals inhibits principal cells in the BA. **(A)** Schematic of the experimental configuration: vCA1 axons expressing ChETA-EYFP were optically stimulated in the BA while recording on-cell from pyramidal neurons (PN). vCA1 axons also synapse on interneurons (IN) in the BA. **(B,C)** Example raster plots **(B1,C1)** and average instantaneous firing rates **(B2,C2)** for BA PNs in response to five, 10-ms light pulses delivered at **(B)** 4 Hz or **(C)** 20 Hz (bins: 50ms; *N = 11* cells). **(D)** Average spike probability for 4 Hz, 20 Hz, and 20 Hz in the presence of the GABAAR antagonist bicuculine (Bic). Data are presented as mean ± SEM.

Stimulation of vCA1 neurons at high-frequencies can produce feed-forward inhibition of principal cells in the BA (Hübner et al., 2014; Bazelot et al., 2015). To examine this possibility, we recorded from BA neurons while stimulating vCA1 terminals at high (20 Hz) or low (4 Hz) frequencies (Figure 5A). AAV5-CaMKII-ChETA was infused into the VH and coronal slices were taken from the BA 2–3 weeks later. BA neurons were excited by 4 Hz stimulation of vCA1 terminals and fired action potentials to every light pulse in the stimulus train (Figures 5B1,B2) (average spike prob ± SEM: pulse 1: 0.97 ± 0.02; pulse 2: 0.88 ± 0.06, pulse 3: 0.86 ± 0.05, pulse 4: 0.86 ± 0.05, pulse 5: 0.91 ± 0.04). In contrast, 20-Hz stimulation only produced a single action potential and suppressed responding to all subsequent pulses (Figures 5C1,C2) (average spike prob. ± SEM: pulse 1: 0.95 ± 0.02; pulse 2: 0.06 ± 0.03, pulse 3: 0.01 ± 0.01, pulse 4: 0.05 ± 0.04, pulse 5: 0.04 ± 0.03) (permutation test, pulses 1–5, 4 Hz vs. 20 Hz: *p* = 0.09, *p* = 0.00, *p* = 0.00, *p* = 0.00, *p* = 0.00). To determine if this suppression was caused by local inhibition, we stimulated vCA1 terminals at 20 Hz in the presence of the GABA_A_ receptor antagonist bicuculine. This manipulation partially rescued the activity of BA neurons (Figure 5D) (average spike prob. ± SEM: pulse 1: 0.96 ± 0.04; pulse 2: 0.72 ± 0.08, pulse 3: 0.25 ± 0.11, pulse 4: 0.09 ± 0.09, pulse 5: 0.05 ± 0.05) (permutation test, pulses 1–5, 20 Hz vs. 20 Hz + Bic: *p* = 0.59, *p* = 0.001, *p* = 0.18, *p* = 0.38, *p* = 0.48), suggesting that feed-forward inhibition plays a role in suppressing excitatory activity when vCA1 neurons are stimulated at high frequencies (Hübner et al., 2014; Bazelot et al., 2015). However, given that firing was not completely rescued, other factors like synaptic depression likely contribute to this effect as well.

### Low Frequency Stimulation of vCA1 Does Not Disrupt the Expression of Context Fear

**FIGURE 6:**
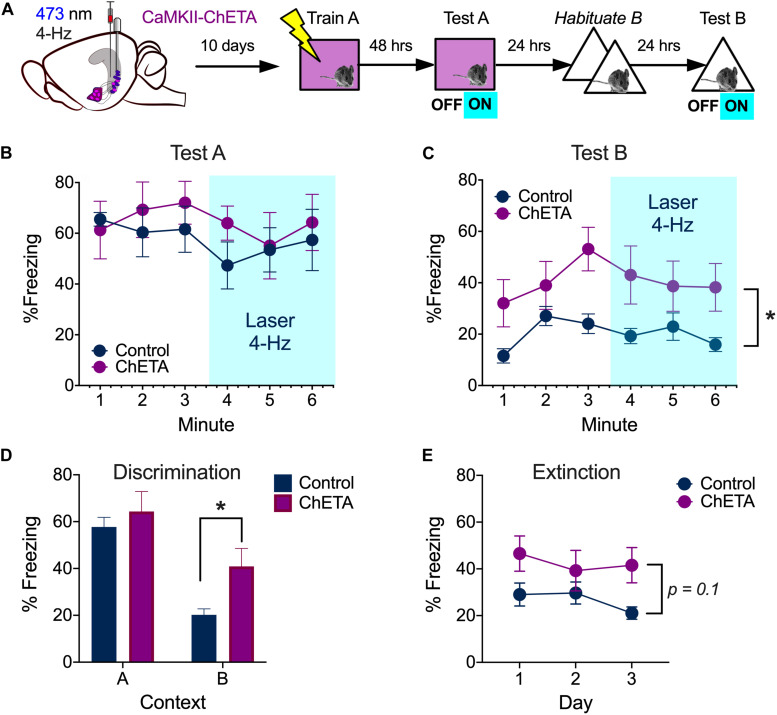
Low frequency stimulation of vCA1 does not disrupt the expression of context fear and enhances generalization. **(A)** Experimental design. **(B)** Percent freezing over time (minutes) during baseline and laser stimulation in the training context (Test A). **(C)** Percent freezing over time (minutes) during baseline and laser stimulation in a novel environment (Test B). **(D)** Percent freezing during the first 3 min of the test in context A and the exposure session in context B 24 h later. **(E)** Percent freezing in control and ChETA groups during the first 3 min of the context B exposure days. All data are presented as the mean ± SEM. *p < 0.05.

During aversive learning, activity in the hippocampus, amygdala and prefrontal cortex becomes synchronized to 4-Hz oscillations (Seidenbecher et al., 2003; Narayanan et al., 2007; Lesting et al., 2011; Padilla-Coreano et al., 2016). Given that BA neurons are able to follow 4 Hz stimulation of vCA1 terminals, we examined the impact of this manipulation on context fear expression. Mice received bilateral infusions of AAV-CaMKII-ChETA-EYFP (*n* = 6) or AAV-CaMKII-EYFP (*n* = 5) into the VH and optic fibers were implanted above vCA1. Two weeks later, they were trained on context fear conditioning as described above. Memory was tested 48 h after training and vCA1 neurons were stimulated at 4 Hz (473 nm, 10 mW, 15 ms pulses) during the last 3 min of the session (Figure 6A, left). Unlike high-frequency stimulation, this manipulation did not disrupt the expression of context fear (Figure 6B) [2-way ANOVA, no laser × virus interaction *F* = 0.05282, *p* = 0.824, no main effect of laser *F*(1, 9) = 0.005282, *p* = 0.8234].

### Fear Generalization Increases After Low-Frequency Stimulation of vCA1 Neurons

We next determined if 4 Hz stimulation could induce freezing in a novel environment. To do this, mice from the previous experiment were first exposed to context B for 2 days to reduce any generalized fear (Figure 6A, right). On day 3, the animals were put back in context B and vCA1 neurons were stimulated at 4 Hz after a 3 min baseline period. This manipulation did not increase freezing in ChETA mice relative to controls (Figure 6C) [2-way repeated measures ANOVA, no Group × Laser interaction, *F*(1, 9) = 0.001, *p* = 0.968]. However, ChETA mice did exhibit an overall increase in freezing in context B, which suggests that prior stimulation altered their behavior [Main effect of Group, *F*(1, 9) = 5.50, *p* = 0.043]. To determine if vCA1 stimulation increased fear generalization, we compared freezing levels during the first exposure session in context B to that observed during the test in context A (Figure 5D). Both ChETA mice and controls froze more in the training context (A) than the novel environment (B), indicating they could discriminate between these places [Two-way ANOVA main effect of context *F*(1, 9) = 76.30, *p* < 0.0001]. Nonetheless, ChETA mice showed significantly more fear in context B than control animals (Bonferroni *post hoc* test, *p* < 0.05), suggesting that vCA1 stimulation increased generalization. However, additional studies will be needed to confirm the selectivity of this effect, as the group × context interaction did not quite reach statistical significance [Two-way repeated measures ANOVA, No group × context interaction *F*(1, 9) = 4.04, *p* = 0.075; no effect of group *F*(1, 9) = 1.94, *p* = 0.19].

Finally, to determine if vCA1 stimulation altered the extinction of generalized fear, we analyzed freezing during the 3 exposure sessions in context B (Figure 6E). Analyses were restricted to the baseline period of each session so we could include the data from day 3. We found that freezing levels decreased slightly across days in both groups, but this change was not statistically significant. This suggests our exposure sessions were not long enough to induce robust extinction [2-way repeated measures ANOVA, No Group × Session interaction *F*(2, 18) = 1.38, *p* = 0.27; No effect of Group *F*(1, 9) = 3.31, *p* = 0.1; No effect of session, *F*(2, 18) = 1.81, *p* = 0.19]. However, it should be noted that the current experiments were not designed to detect small/moderate differences in fear generalization or extinction. Future work will need to use behavioral protocols that are optimized to study these processes in order to determine how they are affected by low-frequency stimulation of vCA1 neurons.

## Discussion

During context fear conditioning, spatial information is thought to be transmitted from dorsal to ventral hippocampus where it can be relayed to the amygdala and associated with shock (Maren and Fanselow, 1995; Wiltgen et al., 2006; Sutherland et al., 2008; Xu et al., 2016; Jimenez et al., 2018; Kim and Cho, 2020). In the current study, we examined the contribution of vCA1 neurons to the expression of context fear. Similar to previous results obtained in the DH, we found that c-Fos expression increased in vCA1 neurons after exposure to a novel environment (Radulovic et al., 1998). However, the addition of shock did not further increase the number of labeled cells as it does in subcortical structures like the amygdala (Milanovic et al., 1998; Radulovic et al., 1998; Barot et al., 2009). This result was surprising given the role of the VH in learned fear and anxiety and the fact that it communicates with subcortical regions involved in emotion (Cenquizca and Swanson, 2006, 2007; Hoover and Vertes, 2007; Jimenez et al., 2018). Nonetheless, it remains possible that footshock activated many of the same cells that responded to the context, making it difficult for us to find a difference between these groups. Consistent with this idea, a recent study showed that vCA1 neurons activated during exploration (c-Fos +) are the same cells that strengthen their connections with BA neurons after the environment is paired with shock (Kim and Cho, 2020).

High-frequency stimulation (20 Hz) of engram cells (c-Fos +) in dorsal DG and CA3 has been shown to increase freezing after context fear conditioning (Ramirez et al., 2013; Ryan et al., 2015; Oishi et al., 2019). However, the same manipulation does not drive freezing when performed in dorsal CA1 (Ryan et al., 2015). We hypothesized that this may be the case because dCA1 neurons do not project to the VH or the amygdala (Fricke and Cowan, 1978; Swanson et al., 1978; Ishizuka et al., 1990). If so, stimulating vCA1 neurons at high frequencies should be able to increase fear. Inconsistent with this prediction, we found that 20 Hz stimulation of BA-projecting vCA1 neurons impaired freezing rather than enhancing it. A similar effect was observed in a previous study when vCA1 terminals in the BA were stimulated at 10 Hz (Jimenez et al., 2018). To determine why high-frequency stimulation produced impairments in freezing, we recorded from principal cells in the BA while activating terminals from vCA1. We found that 20 Hz stimulation inhibited excitatory responses in the BA while 4 Hz did not. The inhibitory effect of 20 Hz stimulation could be reduced with a GABA_A_-receptor antagonist, indicating that it was due, in part, to feed-forward inhibition.

Interestingly, the medial prefrontal cortex (mPFC) has been shown to disinhibit principal cells in the BA and allow them to respond to strong inputs from vCA1 (Hübner et al., 2014). A circuit like this could function to rapidly select adaptive responses in different situations. For example, when animals come across a novel environment place cell activity in vCA1 could inhibit BA neurons and promote exploration. If a threat was subsequently encountered in this same place, input from the mPFC could quickly disinhibit BA neurons and allow vCA1 to drive defensive behaviors like freezing. We plan to examine these ideas in future experiments by simultaneously manipulating inputs from vCA1 and the mPFC to the BA after fear conditioning.

Unlike 20 Hz, stimulation of vCA1 neurons at 4 Hz did not inhibit principal cells in the BA. Given that the hippocampus, amygdala and mPFC oscillate around 4 Hz during fear expression, we hypothesized that low-frequency stimulation may enhance freezing rather than impair it (Seidenbecher et al., 2003; Narayanan et al., 2007; Lesting et al., 2011; Courtin et al., 2014; Karalis et al., 2016; Padilla-Coreano et al., 2016). Consistent with this idea, 4-Hz stimulation increased fear generalization and did not disrupt freezing in the training context. However, freezing did not increase during laser stimulation itself, as has been observed when dCA1 engram cells are stimulated at this frequency (Ryan et al., 2015). It is possible that our behavioral effects would have been larger if we were able to selectively stimulate engram/memory cells in vCA1. In addition, high-frequency stimulation of BA-projecting neurons in vCA1 may be able induce freezing if it co-occurs with a disinhibitory input from the mPFC (Hübner et al., 2014; Karalis et al., 2016).

To summarize, our results suggest that dorsal and ventral CA1 neurons respond similarly to context exploration and fear conditioning. Many cells in each region express c-Fos when animals are exposed to a novel environment, and the number of activated neurons does not increase further if the context is paired with shock. In addition, the expression of fear is impaired when neurons in dorsal or ventral CA1 are stimulated at 20 Hz. Low-frequency stimulation, in contrast, increases freezing and enhances fear generalization in dorsal and ventral CA1, respectively. Additional research will be required to determine if more robust changes in defensive behavior can be induced when firing patterns are coordinated in the mPFC, BA, and vCA1.

## Materials and Methods

### Subjects

Experiments were performed in 2–5-month-old male and female F1 hybrid mice (C57BL/6NT x 129S6/SvEv) from Taconic (B6129F1) or C57BL/6J mice from Jackson Labs (Stock Number #000664). Animals were maintained on a 12 h light/12 h dark cycle with *ad libitum* access to food and water. All experiments were performed during the light portion (7am–7pm) of the light/dark cycle. Mice were group housed until surgery, at which point they were single housed for the remainder of the experiment. All experiments were reviewed and approved by the UC Davis Institutional Animal Care and Use Committee (IACUC).

### Surgeries

Stereotaxic surgery was performed 2–3 weeks before behavioral experiments began. Mice were anesthetized with isoflurane (5% induction, 2% maintenance) and placed in a stereotaxic frame (Kopf Instruments). An incision was made in the scalp and the skull was adjusted to place bregma and lambda in the same horizontal plane. Small holes were drilled above the injection sites for each brain region and virus or tracer was injected through a glass pipette with a tip diameter between 25 and 40 μm using a microsyringe pump (UMP3, World Precision Instruments) at 2 nl/s. In the tracing experiment (Figure 1), Ctb-647 was infused into the BA (50 nl) at each of the following 4 sites (AP, −1.55 mm; ML, ± 2.85; DV, −5 mm and −4.8 mm from dura). In the optogenetic behavioral experiments (Figures 3, 4, 6), AAV-CaMKII ChETA-EYFP, AAV-DIO-ChETA-mCherry or a control virus (AAV-CaMKII-EYFP or AAV-FLEX-TdTomato) were infused into vCA1 (250 nl) at the following 4 sites (AP, −3 mm; ML, ± 3.5 mm; DV −3.9 and −3.5 mm from dura). In Figure 4, AAVrg was also infused into the BA (37 nl) at the following 4 sites (AP, −1.55 mm; ML, ± 2.85; DV, −5.0 mm and −4.9 mm from dura). We waited 3–5 min after each infusion before withdrawing the pipette. Optic fiber cannulas (0.39 NA, 200 μm diameter, Thorlabs) were manufactured as previously described (Sparta et al., 2012) and implanted bilaterally above the virus injection sites in ventral CA1 (AP, –3 mm; ML, ± 3.75 mm, length 3.4 mm). The fibers were secured to the skull using 3 screws and dental cement (Harry J. Bosworth Company). Three- to four-week-old mice were used in the electrophysiology experiments (Figures 2, 5), so the stereotaxic coordinates were adjusted for body size. AAV-ChETA-EYFP (350 nl) was infused into the VHC at the following 2 sites (AP, −2.8 mm; ML, ± 3.6 mm; DV, −2.8 mm from brain surface).

### Contextual Fear Conditioning and Optogenetic Stimulation

Stereotaxic surgery was performed on day 1. On days 8–12, all animals were handled for 5 min a day (either in the vivarium or in a room adjacent to the fear conditioning chambers). Animals in optogenetic experiments had their implants attached to a 1 m split optic patch cable (0.22 NA, 200 μm diameter) during handling. All mice were trained 24 h after the last handling session. Training consisted of 3 min of context exploration and either 2 shocks (0.3 mA, 1 min ITI, Taconic hybrids) or 3 shocks (0.6 mA, 1 min ITI, C57s). Testing consisted of 5-10 min in the training context or a novel context (context B). Mice were sacrificed after training or their final testing session (depending on the experiment) and c-Fos was quantified as described below. For optogenetic experiments, a 473 nm, 300mW DPSS laser system (OptoEngine) was coupled to the branched optic cable and implant through a rotating comutator fixed above the conditioning chamber. Laser output was adjusted to obtain 10 mW from the optic fiber tip measured with an optical power meter (Thorlabs) before each experiment. Doric’s OptG4 software was used to control laser pulse frequency and a Med Associates SG-231 28V DC-TTL adapter was used to control onset and offset of laser pulses during behavioral sessions. Laser stimulation consisted of 3 min epochs with 15 ms pulses at 20 Hz. Mice were trained and/or tested in Med Associates fear conditioning chambers (30.5 × 24.1 × 21.0 cm) that were housed in sound-attenuating boxes containing overhead LED lights and a scanning, charge-coupled video camera. Context A was lit with white light, cleaned with 70% EtOH and contained a stainless-steel grid floor. Context B was lit with infrared light, cleaned with Sani Wipes and contained a smooth plastic insert for the floor (covered with a small amount of corn cob bedding) as well as a curved plastic insert for the walls.

### Immunohistochemistry and Microscopic Imaging

Animals were sacrificed 90 min after training, testing, or final laser stimulation. Mice were deeply anesthetized using 5% isoflurane mixed with O2 and then transcardially perfused with 0.1 M Phosphate buffered saline (PBS) followed by 4% paraformaldehyde (PFA). Brains were extracted and left in PFA for 24–48 h and then sliced into 40 μm coronal sections using a Leica VT-1000 vibratome. To visualize virus spread and locate injection sites and fiber optic tips, two separate series of slices were taken that spanned the anterior-posterior axis (every 5th and 6th slice). For Ctb injection site localization, one series was Nissl-stained and amygdala nuclei were identified using the online Allen interactive mouse reference atlas. The adjacent sections in the other series were stained with DAPI and the location of the injection site was mapped onto the identified Amygdala nuclei. Slices 1–4 were stored for later c-Fos immunohistochemistry. Three to four sections per area were randomly chosen for c-Fos quantification. Slices were incubated in blocking buffer (2% normal Donkey serum, 0.2% Triton-X 100 in 0.1 M PBS) for 15 min followed by overnight incubation in primary antibody at 1:5,000 (Millipore Cat# ABE457, RRID:AB_2631318) suspended in blocking buffer. The next day, tissue was washed 3x with 0.1M PBS and then incubated with a solution containing 1:500 Biotin-SP-conjugated Donkey anti-rabbit secondary antibody (Jackson ImmunoResearch Labs Cat# 711-065-152, RRID: AB_2340593). After washing, the antibodies were detected using Streptavidin-conjugated Cy3 (1:500), (Jackson ImmunoResearch Labs Cat# 016-160-084, RRID: AB_2337244) or Cy5 (1:250), (Jackson ImmunoResearch Labs Cat# 016-170-084, RRID: AB_2337245). Finally, sections were counterstained with DAPI (1:10,000, Life Technologies) for 15 min and mounted on slides (Vectashield mounting medium). Slides were imaged using an Olympus fluorescence virtual slide scanning microscope. For c-Fos quantification, 35 μm z-stacks were acquired at 20x magnification. ROIs were chosen in vCA1 either beneath the optic fiber tip (optogenetic experiments) or in sections that contained AMY projecting neurons (ctb experiment). Fluorescent images were imported into FIJI, converted to grayscale and separated by channel. Fluorescent label was marked on each channel independently using the FIJI cell counter tool and the macro metamorph emulator (©2005 Fabrice P. Cordelières). Overlap (as in ctb experiment) was determined by superimposing the markers from one channel onto another and counting the number of overlapping markers. For any experiments estimating the percent of cells expressing label out of the total number of cells per area, the 3D Objects Counter tool in FIJI was used to estimate the number of DAPI stained nuclei in each area by dividing the obtained volume by the average single nucleus volume for the animal/area. For quantification of c-fos in all dorsal and ventral CA1 neurons (Figures 1F,G), 35 μm single-plane images were acquired at 20x magnification. Images were cropped to contain approximately 10,000 μm^2^ of area CA1 in both the dorsal and ventral hippocampus. After acquisition, images were converted to grayscale and c-Fos positive cells were quantified using the FIJI cell counter tool.

### Virus Constructs

The following constructs (AAV2, serotype 5) were packaged by the Vector Core at the University of North Carolina: AAV5 -CaMKII-hChR2(E123T/T159C)-p2A-EYFP-WPRE had a titer of 3.6 × 10^12^ – 4.1 × 10^12^ viral particles/ml. AAV5- EF1a-DIO-hChR2 (E123T/T159C) p2A-mCherry had a titer of 4.10e^12^ virus molecules/ml. AAV5-CAG-FLEX-tdTomato had a titer of 4.8e^12^ viral particles/ml. The AAVrg-cre-EBFP plasmid was purchased from Addgene (catalog# 51507) and packaged by the UC Davis Vector Core with a titer of 7.63^12^ GC/ml.

### Slice Preparation for Electrophysiological Recordings

Mice (postnatal week 6-7; both sexes) were anesthetized through intraperitoneal injection of an anesthetic cocktail (ketamine: 10 mg/kg; xylazine: 1 mg/kg; acepromazine: 0.1 mg/kg) and transcardially perfused with ice-cold artificial CSF (aCSF; in mM: 127 NaCl, 2.5 KCl, 1.25 NaH_2_PO_4_, 25 NaHCO_3_, 1 MgCl_2_, 2 CaCl_2_, 25 glucose; supplemented with 0.4 sodium ascorbate and 2 sodium pyruvate; ∼310 mOsm). Brains were rapidly removed, blocked, and placed in choline slurry (110 choline chloride, 25 NaHCO_3,_ 25 glucose, 2.5 KCl, 1.25 NaH_2_PO_4_, 7 MgCl_2_, 0.5 CaCl_2_, 11.6 sodium ascorbate, 3.1 sodium pyruvate; ∼310 mOsm). Coronal sections (250 μm) containing vCA1 or BA were cut on a vibratome (Leica VT1200S) and transferred to an incubation chamber containing aCSF at 32°C for 25 min before moving to room temperature until used for recordings. All solutions were bubbled with 95% O_2_–5% CO_2_ continuously. Chemicals were from Sigma.

### Patch-Clamp Recordings

For recordings, slices were mounted onto glass coveslips coated with poly-l-lysine and placed in a submersion chamber perfused with aCSF (2 ml/min) at 30–32°C. Loose on-cell patch-clamp recordings were made from visually identified cells in vCA1 or BAusing borosilicate glass pipettes (3–5 MΩ) filled with 150 mM NaCl. This configuration does not perturb the intracellular milieu of the recording cell. vCA1 pyramidal neurons were identified based on position and shape and were selected for ChETA-EYFP expression. BA primary neurons (PNs) were identified based on size (>15 μm) and firing rate (<20 Hz) (Sosulina et al., 2006; Bazelot et al., 2015). Recordings were performed in voltage clamp mode by setting the pipette potential to obtain 0 pA of membrane current (Perkins, 2006) and were acquired in pClamp11 using a Multiclamp 700B amplifier (Molecular Devices). Recordings were digitized at 20 kHz with a Digidata 1550 digitizer (Molecular Devices), and low-pass filtered at 8 kHz. Optical stimulation of ChETA-expressing hippocampal pyramidal neurons in vCA1 or their axons in BA was performed under a 60x water immersion lens (1.0 N.A.) of an Olympus BX51W microscope, using an LED system (Prizmatix UHP or Excelitas X-cite; max power of 3 mW at lens tip) mounted on the microscope and driven by a Master9 stimulator (AMPI). Stimulation consisted of 5 10 ms pulses of 488 nm light delivered at various frequencies, as indicated. Each protocol was repeated at least 5 times per stimulation frequency with an inter-trial interval of 30 s [to allow for opsin recovery (Lin, 2011)]. Pulses of increasing power were delivered until an action potential was triggered. Above threshold values (∼1.5–2x threshold) were used for experiments. For vCA1 axonal stimulation in BA, higher values were also tested to examine whether more than 1 spikes could be synaptically evoked in BAPNs at 20 Hz. The GABA_A_ receptor blocker bicuculine (20 μM) was washed in during BArecordings, as indicated, for 6 min before resuming stimulation.

### Data Analysis

For the behavioral experiments, group differences were analyzed with ANOVAs and Bonferroni *post hoc* tests. All statistics were done using GraphPad Prism (2018).

For electrophysiology experiments, data were analyzed with custom-made tools in MATLAB (Mathworks). Spike probability was quantified as the probability of an action potential being evoked during repetitions of the same stimulation regime. For vCA1 recordings, an action potential was considered as evoked if it occurred within a time window of 10 ms from pulse onset (i.e., during the pulse). For BA PN recordings, an action potential was considered as evoked if it occurred within a time window of 15 ms from pulse onset (the longer time window was used to account for synaptic delays). Baseline spike probability was quantified as the average probability of an action potential within 500 randomly selected time windows (10 ms for vCA1; 15 ms for BA) during the 3-s pre-stimulus baseline. For peri-stimulus time histograms (PSTH), action potentials were counted in 50-ms bins, with time referenced to the start of light pulses.

Permutation tests were used for statistical comparisons of average spike probabilities between conditions (Odén and Wedel, 1975). Data were randomly shuffled between conditions 1,000 times, while maintaining the original sample sizes, and the differences between the group averages of observed spike probabilities were compared against the corresponding differences between the group averages of random permutations. The reported p-values indicate the probability that a difference between average spike probabilities equal to or greater than the observed difference could have arisen by chance alone (i.e., due to random sampling).

## Data Availability Statement

The raw data supporting the conclusions of this article will be made available by the authors, without undue reservation.

## Ethics Statement

The animal studies were reviewed and approved by the UC Davis, IACUC.

## Author Contributions

The behavioral experiments were designed by JG and BW, performed by JG, YTO, NV, and RV and analyzed by JG, YTO, and BW. DF designed the electrophysiology experiments. DF, AD, AP, and SJJ performed the electrophysiology experiments. DF and EA analyzed the electrophysiology experiments. JG, DF, and BW wrote the manuscript. All authors have seen and approved this manuscript.

## Conflict of Interest

The authors declare that the research was conducted in the absence of any commercial or financial relationships that could be construed as a potential conflict of interest.

## References

[B1] Balogh S. A. Wehner J. A. (2003). Inbred mouse strain differences in the establishment of long-term fear memory. *Behav. Brain Res*. 140 97–106. 10.1016/s0166-4328(02)00279-6 12644283

[B2] Barot S. K. Chung A. Kim J. J. Bernstein I. L. (2009). Functional imaging of stimulus convergence in amygdalar neurons during pavlovian fear conditioning. *PLoS One* 4:e6156. 10.1371/journal.pone.0006156 19582153 PMC2701998

[B3] Bazelot M. Bocchio M. Kasugai Y. Fischer D. Dodson P. D. Ferraguti F. (2015). Hippocampal theta input to the amygdala shapes feedforward inhibition to gate heterosynaptic plasticity. *Neuron* 87 1290–1303. 10.1016/j.neuron.2015.08.024 26402610 PMC4590554

[B4] Berndt A. Schoenenberger P. Mattis J. Tye K. M. Deisseroth K. Hegemann P. (2011). High-efficiency channelrhodopsins for fast neuronal stimulation at low light levels. *PNAS* 108 7595–7600. 21504945 10.1073/pnas.1017210108 PMC3088623

[B5] Beyeler A. Chang C. Silvestre M. Leveque C. Namburi P. Wildes C. P. (2018). Organization of valence-encoding and projection-defined neurons in the basolateral amygdala. *Cell Rep*. 22 905–918. 10.1016/j.celrep.2017.12.097 29386133 PMC5891824

[B6] Cenquizca L. A. Swanson L. W. (2006). Analysis of direct hippocampal cortical field ca1 axonal projections to diencephalon in the rat. *J. Comp. Neurol*. 497 101–114. 10.1002/cne.20985 16680763 PMC2570652

[B7] Cenquizca L. A. Swanson L. W. (2007). Spatial organization of direct hippocampal field CA1 axonal projections to the rest of the cerebral cortex. *Brain Res. Rev*. 56 1–26. 10.1016/j.brainresrev.2007.05.002 17559940 PMC2171036

[B8] Ciocchi S. Passecker J. Malagon-Vina H. Mikus N. Klausberger T. (2015). Selective information routing by ventral hippocampal ca1 projection neurons. *Science* 348 560–563. 10.1126/science.aaa3245 25931556

[B9] Courtin J. Chaudun F. Rozeske R. R. Karalis N. Gonzalez-Campo C. Wurtz H. (2014). Prefrontal parvalbumin interneurons shape neuronal activity to drive fear expression. *Nature* 505 92–96. 10.1038/nature12755 24256726

[B10] Crestani A. P. Krueger J. N. Barragan E. V. Nakazawa Y. Nemes S. E. Quillfeldt J. A. (2019). Metaplasticity contributes to memory formation in the hippocampus. *Neuropsychopharmacology* 44 408–414. 10.1038/s41386-018-0096-7 29849054 PMC6300591

[B11] Fanselow M. S. Dong H. W. (2010). Are the dorsal and ventral hippocampus functionally distinct structures? *Neuron* 65 7–19. 10.1016/j.conb.2013.11.010 20152109 PMC2822727

[B12] Fricke R. Cowan W. M. (1978). An autoradiographic study of the commissural and ipsilateral hippocampo-dentate projections in the adult rat. *J. Comp. Neurol*. 181 253–269. 10.1002/cne.901810204 567658

[B13] Graham J. D’Ambra A. Jung S. J. Vishwakarma N. Venkatesh R. Parigi A. (2020). High frequency stimulation of ventral CA1 neurons reduces amygdala activity and inhibits fear. *BioRxiv [Preprint]*. 10.1101/2020.07.01.183210 PMC7985556 33767614

[B14] Hess U. S. Gary L. Gall C. M. (1995). Regional patterns of C-Fos MRNA expression in rat hippocampus following exploration of a novel environment versus performance of a well-learned discrimination. *J. Neurosci*. 15 7796–7809. 10.1523/jneurosci.15-12-07796.1995 8613720 PMC6577969

[B15] Hoover W. B. Vertes R. P. (2007). Anatomical analysis of afferent projections to the medial prefrontal cortex in the rat. *Brain Struct. Funct*. 212 149–179. 10.1007/s00429-007-0150-4 17717690

[B16] Hübner C. Bosch D. Gall A. Lüthi A. Ehrlich I. (2014). Ex vivo dissection of optogenetically activated mPFC and hippocampal inputs to neurons in the basolateral amygdala: implications for fear and emotional memory. *Front. Behav. Neurosci*. 8:64. 10.3389/fnbeh.2014.00064 24634648 PMC3943336

[B17] Ishizuka N. Weber J. Amaral D. G. (1990). Organization of intrahippocampal projections originating from CA3 pyramidal cells in the rat. *J. Comp. Neurol*. 295 580–623. 10.1002/cne.902950407 2358523

[B18] Jimenez J. C. Su K. Goldberg A. R. Luna V. M. Biane J. S. Ordek G. (2018). Anxiety cells in a hippocampal-hypothalamic circuit. *Neuron* 97 670–683. 10.1016/j.neuron.2018.01.016 29397273 PMC5877404

[B19] Karalis N. Dejean C. Chaudun F. Khoder S. Rozeske R. Wurtz H. (2016). 4 Hz oscillations synchronize prefrontal–amygdala circuits during fear behaviour. *Nat. Neurosci*. 19 605–612. 10.1038/nn.4251.4 26878674 PMC4843971

[B20] Kim W. B. Cho J. H. (2020). Encoding of contextual fear memory in hippocampal–amygdala circuit. *Nat. Commun*. 11 1–22. 10.1038/s41467-020-15121-2 32170133 PMC7069961

[B21] Kjelstrup K. B. Solstad T. Brun V. H. Hafting T. Leutgeb S. Witter M. P. (2008). Finite scale of spatial representation in the hippocampus. *Science* 321 140–143. 10.1126/science.1157086 18599792

[B22] Kjelstrup K. G. Tuvnes F. A. Steffenach H. Murison R. Moser E. I. Moser M. B. (2002). Reduced fear expression after lesions of the ventral hippocampus. *Proc. Natl. Acad. Sci. U.S.A*. 99 10825–10830. 10.1073/pnas.152112399 12149439 PMC125057

[B23] Komorowski R. W. Garcia C. G. Wilson A. Hattori S. Howard M. W. Eichenbaum H. (2013). Ventral hippocampal neurons are shaped by experience to represent behaviorally relevant contexts. *J. Neurosci*. 33 8079–8087. 10.1523/JNEUROSCI.5458-12.2013 23637197 PMC3667351

[B24] Krueger J. N. Wilmot J. H. Teratani-Ota Y. Puhger K. R. Nemes S. E. Crestani A. P. (2020). Amnesia for context fear is caused by widespread disruption of hippocampal activity. *Neurobiol. Learn. Mem.* 175:107295. 10.1016/j.nlm.2020.107295 32822864 PMC8562570

[B25] Lesting J. Narayanan R. T. Kluge C. Sangha S. Seidenbecher T. Pape H. C. (2011). Patterns of coupled theta activity in amygdala-hippocampal-prefrontal cortical circuits during fear extinction. *PLoS One* 6:e21714. 10.1371/journal.pone.0021714 21738775 PMC3125298

[B26] Lin J. Y. (2011). A user’s guide to channelrhodopsin variants: features, limitations and future developments. *Exp. Physiol.* 96 19–25. 10.1113/expphysiol.2009.051961 20621963 PMC2995811

[B27] Lovett-Barron M. Kaifosh P. Kheirbek M. A. Danielson N. Zaremba J. D. Reardon T. R. (2014). Dendritic inhibition in the hippocampus suports fear learning. *Science* 343 857–864. 10.1126/science.1247485 24558155 PMC4018419

[B28] Maren S. Fanselow M. S. (1995). Synaptic plasticity in the basolateral amygdala induced by hippocampal formation stimulation in vivo. *J. Neurosci*. 15 7548–7564. 7472506 10.1523/JNEUROSCI.15-11-07548.1995 PMC6578043

[B29] Milanovic S. Radulovic J. Laban O. Stiedl O. Henn F. Spiess J. (1998). Production of the fos protein after contextual fear conditioning of C57BL/6N mice. *Brain Res*. 784 37–47. 9518543 10.1016/s0006-8993(97)01266-3

[B30] Moser E. I. Moser M. B. McNaughton B. L. (2017). Spatial representation in the hippocampal formation: a history. *Nat. Neurosci*. 20 1448–1464. 10.1038/nn.4653 29073644

[B31] Moser M. B. Moser E. I. (1998). Functional differentiation in the hippocampus. *Hippocampus* 8 608–619. 10.1002/(SICI)1098-106319988:6<608::AID-HIPO3<3.0.CO;2-7 9882018

[B32] Nakazawa Y. Pevzner A. Tanaka K. Z. Wiltgen B. J. (2016). Memory retrieval along the proximodistal axis of CA1. *Hippocampus* 26 1140–1148. 10.1002/hipo.22596 27068122 PMC4996732

[B33] Narayanan R. T. Seidenbecher T. Kluge C. Bergado J. Stork O. Pape H. C. (2007). Dissociated theta phase synchronization in amygdalo-hippocampal circuits during various stages of fear memory. *Eur. J. Neurosci*. 25 1823–1831. 10.1111/j.1460-9568.2007.05437.x 17408428

[B34] Odén A. Wedel H. (1975). Arguments for fisher’s permutation test. *Ann. Stat*. 3 518–520.

[B35] Oishi N. Nomoto M. Ohkawa N. Saitoh Y. Sano Y. Tsujimura S. (2019). Artificial association of memory events by optogenetic stimulation of hippocampal CA3 cell ensembles. *Mol. Brain* 12:2. 10.1186/s13041-018-0424-1 30621738 PMC6323779

[B36] O’Keefe J. Dostrovsky J. (1971). The hippocampus as a spatial map. preliminary evidence from unit activity in the freely-moving rat. *Brain Res*. 34 171–175. 5124915 10.1016/0006-8993(71)90358-1

[B37] O’Keefe J. Speakman A. (1987). Single unit activity in the rat hippocampus during a spatial memory task. *Exp. Brain Res*. 68 1–27. 3691688 10.1007/BF00255230

[B38] Owen E. H. Logue S. F. Rasmussen D. L. Wehner J. M. (1997). Assessment of learning by the morris water task and fear conditioning in inbred mouse strains and F1 hybrids: implications of genetic background for single gene mutations and quantitative trait loci analyses. *Neuroscience* 80 1087–1099. 10.1016/S0306-4522(97)00165-6 9284062

[B39] Padilla-Coreano N. Bolkan S. S. Pierce G. M. Dakota R. B. Hardin W. D. Garcia-Garcia A. L. (2016). Direct ventral hippocampal-prefrontal input is required for anxiety-related neural activity and behavior. *Neuron* 89 857–866. 10.1016/j.neuron.2016.01.011 26853301 PMC4760847

[B40] Pelletier J. G. Likhtik E. Filali M. Paré D. (2005). Lasting increases in basolateral amygdala activity after emotional arousal: implications for facilitated consolidation of emotional memories. *Learn. Mem*. 12 96–102. 10.1101/lm.88605.2 15805308 PMC1074326

[B41] Perkins K. L. (2006). Cell-attached voltage-clamp and current-clamp recording and stimulation techniques in brain slices. *J. Neurosci. Methods* 154 1–18. 10.1016/j.jneumeth.2006.02.010 16554092 PMC2373773

[B42] Radulovic J. Kammermeier J. Joachim S. (1998). Relationship between fos production and classical fear conditioning: effects of novelty, latent inhibition, and unconditioned stimulus preexposure. *J. Neurosci*. 18 7452–7461. 9736664 10.1523/JNEUROSCI.18-18-07452.1998 PMC6793227

[B43] Ramirez S. Liu X. Lin P. A. Suh J. Pignatelli M. Redondo R. L. (2013). Creating a false memory in the hippocampus. *Science* 341 387–391. 10.1126/science.1239073 23888038

[B44] Ryan T. J. Roy D. S. Pignatelli M. Arons A. Tonegawa S. (2015). Engram cells retain memory under retrograde amnesia. *Science* 348 1007–1014. 26023136 10.1126/science.aaa5542 PMC5583719

[B45] Seidenbecher T. Laxmi R. T. Stork O. Pape H. C. (2003). Amygdalar and hippocampal theta rhythm synchronization. *Science* 846 846–851. 10.1126/science.1085818 12907806

[B46] Sosulina L. Meis S. Seifert G. Steinhauser C. Pape H. C. (2006). Classification of projection neurons and interneurons in the rat lateral amygdala based upon cluster analysis. *Mol. Cell Neurosci.* 33 57–67. 10.1016/j.mcn.2006.06.005 16861000

[B47] Sparta D. R. Stamatakis A. M. Phillips J. L. Hovelsø N. van Zessen R. Stuber G. D. (2012). Construction of implantable optical fibers for long-term optogenetic manipulation of neural circuits. *Nat. Protoc*. 7 12–23. 10.1038/nprot.2011.413 22157972 PMC8647635

[B48] Strange B. A. Witter M. P. Lein E. S. Moser E. I. (2014). Functional organization of the hippocampal longitudinal axis. *Nat. Publ. Group* 15 655–669. 10.1038/nrn3785 25234264

[B49] Sutherland R. J. O’Brien J. Lehmann H. (2008). Absence of systems consolidation of fear memories after dorsal, ventral, or complete hippocampal damage. *Hippocampus* 18 710–718. 10.1002/hipo.20431 18446823

[B50] Swanson L. W. Wyss J. M. Cowan W. M. (1978). An autoradiographic study of the organization of intrahippocampal association pathways in the rat. *J. Comp. Neurol*. 181 681–715. 10.1002/cne.901810402 690280

[B51] Tanaka K. Z. Pevzner A. Hamidi A. B. Nakazawa Y. Graham J. Wiltgen B. J. (2014). Cortical representations are reinstated by the hippocampus during memory retrieval. *Neuron* 84 347–357. 10.1016/j.neuron.2014.09.037 25308331

[B52] Tanaka K. Z. He H. Anupratap T. Niisato K. Huang A. J. Y. McHugh T. J. (2018). The hippocampal engram maps experience but not place. *Science* 361 392–397. 10.1126/science.aat5397 30049878

[B53] Tayler K. K. Tanaka K. Z. Reijmers L. G. Wiltgen B. J. (2013). Reactivation of neural ensembles during the retrieval of recent and remote memory. *Curr. Biol.* 23 99–106. 10.1016/j.cub.2012.11.019 23246402

[B54] Wilmot J. H. Graham J. A. LaFreniere M. M. Puhger K. Wiltgen B. J. (2018). “Altered immediate early gene expression in fos-tTA transgenic mice,” in *Proceedings of the Society for Neuroscience Meeting*, San Diego, CA. Program No. (331.26), Session No. (HHH49).

[B55] Wilmot J. H. Puhger K. Wiltgen B. J. (2019). Acute disruption of the dorsal hippocampus impairs the encoding and retrieval of trace fear memories. *Front. Behav. Neurosci*. 13:116. 10.3389/fnbeh.2019.00116 31191269 PMC6548811

[B56] Wiltgen B. J. Sanders M. J. Anagnostaras S. G. Sage J. R. Fanselow M. S. (2006). Context fear learning in the absence of the hippocampus. *J. Neurosci*. 26 5484–5491. 10.1523/JNEUROSCI.2685-05.2006 16707800 PMC6675287

[B57] Wolff S. B. E. Gründemann J. Tovote P. Krabbe S. Jacobson G. A. Müller C. (2014). Amygdala interneuron subtypes control fear learning through disinhibition. *Nature* 509 453–458. 10.1038/nature13258 24814341

[B58] Xu C. Krabbe S. Gründemann J. Botta P. Fadok J. P. Osakada F. (2016). Distinct hippocampal pathways mediate dissociable roles of context in memory retrieval. *Cell* 167:961-972.e16. 10.1016/j.cell.2016.09.051 27773481 PMC5382990

[B59] Yizhar O. Fenno L. E. Davidson T. J. Mogri M. Deisseroth K. (2011). Optogenetics in neural systems. *Neuron* 71 9–34. 10.1016/j.neuron.2011.06.004 21745635

